# Outcomes and patterns of care of patients with locally advanced oropharyngeal carcinoma treated in the early 21^st^ century

**DOI:** 10.1186/1748-717X-8-21

**Published:** 2013-01-29

**Authors:** Adam S Garden, Merrill S Kies, William H Morrison, Randal S Weber, Steven J Frank, Bonnie S Glisson, Gary B Gunn, Beth M Beadle, K Kian Ang, David I Rosenthal, Erich M Sturgis

**Affiliations:** 1 Department of Radiation Oncology, University of Texas M.D. Anderson Cancer Center, 1515 Holcombe Blvd, Houston, TX 77030, USA; 2 Department of Thoracic/Head and Neck Medicine, University of Texas M. D. Anderson Cancer Center, 1515 Holcombe Blvd, Houston, TX 77030, USA; 3 Department of Head and Neck Surgery, University of Texas M. D. Anderson Cancer Center, 1515 Holcombe Blvd, Houston, TX 77030, USA; 4 Department of Epidemiology, University of Texas M.D. Anderson Cancer Center, 1515 Holcombe Blvd, Houston, TX 77030, USA

**Keywords:** Radiation, Oropharyngeal cancer, IMRT, Chemoradiation, Squamous cell

## Abstract

**Background:**

We performed this study to assess outcomes of patients with oropharyngeal cancer treated with modern therapy approaches.

**Methods:**

Demographics, treatments and outcomes of patients diagnosed with Stage 3- 4B squamous carcinoma of the oropharynx, between 2000 – 2007 were tabulated and analyzed.

**Results:**

The cohort consisted of 1046 patients. The 5- year actuarial overall survival, recurrence-free survival and local-regional control rates for the entire cohort were 78%, 77% and 87% respectively. More advanced disease, increasing T-stage and smoking were associated with higher rates of local-regional recurrence and poorer survival.

**Conclusions:**

Patients with locally advanced oropharyngeal cancer have a relatively high survival rate. Patients’ demographics and primary tumor volume were very influential on these favorable outcomes. In particular, patients with small primary tumors did very well even when treatment was not intensified with the addition of chemotherapy.

## Introduction

During the latter part of the 20^th^ century, several changes occurred in the management and epidemiology of head and neck cancer. Numerous trials were conducted investigating intensification of therapy. One avenue of investigation was altered fractionation of radiation schedules. Multiple trials demonstrated a benefit to mildly accelerating radiation schedules, or hyperfractionating radiation [1-3]. Incorporation of chemotherapy to improve disease control and allow for organ preservation was studied extensively during 1980 – 2000 [4]. Concomitant chemotherapy and radiation has become established as a standard of non-surgical care for patients with locally advanced disease. Sequential induction chemotherapy followed by definitive radiotherapy, with or without concomitant chemotherapy, remains under study; however, there has been FDA approval for use of docetaxel, cisplatin and fluorouracil (TPF) as an induction regimen in selected patients [5].

Intensity-modulated radiation therapy (IMRT) also was developed during the last decade of the 20^th^ century. IMRT is a system of radiation treatment planning and delivery that allows for more optimal radiation dose distributions. Favorable early reports published in the first few years of the past decade [6-8] led to the incorporation of IMRT into many cooperative group trials, and there has been a striking increase between 2000–2010 in the use of IMRT as a routine therapy for head and neck cancer [9].

These changes in management have paralleled a change in the epidemiology of head and neck cancer in the past 2 decades, and particularly oropharyngeal cancer. There has been a dramatic increase in the incidence of oropharyngeal cancer particularly among middle-aged white men [10]. With declining smoking prevalence over this timeframe, the phenomenon of rising oropharyngeal cancer incidence has been attributed to the prevalence of oropharyngeal cancer associated with human papillomavirus (HPV) [11,12]. Retrospective series and, more recently, secondary analyses of prospective clinical trials have demonstrated better prognoses for patients with HPV positive disease compared with similarly treated patients who are HPV negative [13-15].

In tandem with the therapeutic advances described above, we progressively intensified therapy for patients with oropharyngeal carcinoma, though we often attempted to use a risk based approach [16] that incorporated disease volume and location rather than uniformly deliver identical therapy for all stage 3 and 4 patients. Previous reports from our group suggested that patients with multiple nodes or nodal disease in levels 3 and 4 had a greater risk of developing distant disease [17]. In general, we favored neoadjuvant therapy for these patients in attempt to reduce distant metastasis risk. Decisions for adding concurrent chemotherapy were based more often on T-category, with higher staged patients treated with greater therapy intensification. As our management approach evolved, we observed demographic changes in our patients similar to those occurring on a national level. This study was conducted to assess our patients’ outcomes and determine what factors were the most influential.

## Methods

The database maintained by the Department of Radiation Oncology at The University of Texas M.D. Anderson Cancer Center (MDACC) was searched to identify patients irradiated for oropharyngeal carcinoma (squamous cell, poorly differentiated or undifferentiated, or not otherwise specified) between the years 2000–2007. Our institutional review board granted permission to conduct this retrospective study.

The search identified 1162 medical records. Patients were excluded for the following reasons: distant metastases or concurrent malignancies (exclusive of a second malignancy of the oropharynx) at the time of diagnosis (16 patients), a previously treated malignancy of the head and neck or previous radiation to the head or neck (8), a history of any malignancy (excluding non-melanomatous skin cancer) within two years of diagnosis (7), or treatment with chemotherapy prior to staging at MDACC (8). In addition 69 patients who did not meet the staging criteria of interest (Stage 3- 4B), and 8 patients with poor performance statuses, staged 4B, and treated with palliative intent were excluded. One thousand forty-six patients formed the cohort for analysis.

Medical records were reviewed to assess patients’ demographic, clinical, radiologic and pathologic data. Based upon the medical history at presentation and as described previously [18] patients were classified as current smokers, former smokers, or never-smokers. Smokers were further evaluated to assess if they quit smoking, or continued to smoke during or subsequent to treatment.

Patients’ disease was staged according to the AJCC 2002 staging system [19]. Charts were reviewed to verify tumor size and sites of invasion. Staging variables of interest included T-category, N-category, and overall AJCC group stage. Patients staged Tx were typically those seen post-tonsillectomy and if the tumor size could not be determined after record review, these patients were staged T1 for the purpose of AJCC stage grouping in this analysis. Those staged Nx were patients in whom a solitary node was excised for diagnosis, and size could not be determined. These patients were coded as N1 for the purpose of this analysis.

Chi-squared tests were used to compare proportions between subsets. The t-test was used for comparison of means. The Kaplan-Meier method was used to calculate actuarial curves. Time of diagnosis was used as time zero. Comparisons between survival curves were made using the log-rank test. Multivariate analysis was performed using the Cox proportional model.

Our approach has been to perform neck dissection only in patients with suspected residual disease following radiation. During the years of this study reassessment principally consisted of physical examination and CT scan 6 to 8 weeks after radiation. Those patients with an obvious residual mass were operated. Patients with questionable residual disease had sonograms with aspiration performed to try to resolve whether there was viable disease. Routine use of positron-emission tomography had not become a routine practice during the years of this study. Details of our experience with regards to management of the neck in an overlapping cohort has been recently described [20]. Patients who had neck dissections performed within 6 months of radiation for suspected residual disease were not scored as having disease recurrence.

## Results

### Demographics and staging

Table 1 details the T and N stages of the 1046 patients. Despite having “locally advanced” head and neck cancer, 62% of patients had T1-T2 tumors. Identification as having stage 3-4B disease was often based on the presence of nodal disease, as only 5% of patients were node negative.

**Table 1:** T and N stages of 1046 patients with stage 3- 4b oropharyngeal cancer

		**N-category**						**Total**
		**0**	**1**	**2a**	**2b**	**2c**	**3**	
T-category	1	0	81	69	118	26	22	316
	2	0	59	47	134	53	31	324
	3	37	30	6	81	46	19	219
	4A	13	18	3	36	59	17	146
	4B	7	4	0	10	10	10	41
Total		57	192	125	379	194	99	1046

Patients’ demographics, tumor sites and staging are detailed in Table 2. Never smokers comprised 41% of the cohort. Former smokers had quit 1 – 53 years prior to diagnosis (median, 18 years). Among all smokers, the median and mean pack years were 30 and 34, though there was a difference between former and current smokers, with mean pack years of 27 and 45, respectively. Thirty-one percent of former smokers, 56% of current smokers who quit at diagnosis, and 78% of smokers who continued to smoke had >30 pack year history at diagnosis (p < .001). The tonsil and base of tongue were the most common primary sites. Tonsil and base of tongue primary sites accounted for only 85% of current smoking patients compared with 94% and 96% for former and never smokers, respectively (p < .001). There were also significant differences among the stages of the 3 smoking groups. The overall group staging was different among the smoking groups as Stage 4B was most common among current smokers (p < .001). Never smokers had a greater proportion of smaller primary tumors (71%, T1-2) compared with former smokers (62%, T1-2), and current smokers (44%, T1-T2). Differences in N-category among the 3 smoking groups were not statistically significant, though the trends observed were for never smokers to have a higher proportion of N1-2c patients and current smokers to have a higher proportion of N0 and N3 patients.

**Table 2:** Patient demographics and treatment

	**All (*N *= 1046) No. (%)**	**Current smokers (*N *= 242) No. (%)**	**Former smokers (*N *= 381) No. (%)**	**Never smokers (*N *= 423) No. (%)**	***p*- value**
Age in years					
Mean (range)	56.2 (28–87)	55.6 (35 – 80)	59.3 (36 – 87)	53.7 (28 – 81)	.01
Median	55	55	58	53	
Sex					.725
Male	906 (87)	207 (86)	334 (88)	365 (86)	
Female	140 (13)	35 (14)	47 (12)	58 (14)	
Race					<.001
White	930 (89)	197 (21)	353 (38)	380 (41)	
Black	47 (5)	29 (62)	3 (6)	15 (32)	
Hispanic	55 (5)	12 (22)	19 (35)	24 (44)	
Other	14 (1)	5 (36)	6 (43)	3 (21)	
Cigarette pack-year					<.001
None	423 (42)			423 (100)	
1 - 30	339 (32)	86 (36)	253 (69)		
>30	265 (26)	153 (64)	112 (31)		
Primary site					<.001
Tonsil	460 (44)	111 (46)	171 (45)	178 (42)	
Base of tongue	511 (49)	95 (39)	186 (49)	230 (54)	
Other*	75 (7)	36 (15)	24 (6)	15 (4)	
T-category					<.001
1-2	640 (62)	107 (44)	235 (62)	298 (71)	
3-4	406 (38)	135 (56)	146 (38)	125 (30)	
N-category					.07
0	57 (5)	20 (8)	22 (6)	15 (4)	
1 -2a	317 (30)	63 (26)	120 (31)	134 (32)	
2b – 2c	573 (55)	128 (53)	205 (54)	240 (57)	
3	99 (9)	31 (13)	34 (9)	34 (8)	
Stage					.001
3	206 (20)	43 (18)	83 (22)	80 (19)	
4A	707 (68)	149 (62)	257 (68)	30 (72)	
4B	133 (13)	50 (21)	41 (11)	42 (10)	
Treatment					.176
XRT alone	415 (40)	83 (34)	158 (42)	175 (41)	
CTXRT	389 (37)	102 (43)	146 (38)	141 (33)	
Ind. CTX > XRT	118 (11)	29 (12)	35 (9)	53 (13)	
Ind. CTX > CTXRT	124 (12)	28 (12)	42 (11)	54 (14)	
Radiation technique					<.001
IMRT	714 (69)	141 (58)	211 (69)	316 (75)	
3D conformal	328 (31)	101 (42)	120 (31)	107 (25)	
Radiation Fractionation					.442
Once- daily	700 (67)	154 (64)	250 (66)	296 (70)	
Altered fractionation	346 (33)	88 (36)	131 (34)	127 (30)	

*Other primary sites: soft palate, 19 patients; pharyngeal wall, 34, NOS (not otherwise specified), 22.

XRT – Radiotherapy; CTXRT – concurrent chemoradiation; Ind. CTX – Induction chemotherapy.

Nodal location correlated with nodal stage. Only 4% of patients with stage N1-2a had nodes in levels 3 or 4 compared with 51% of patients staged N2b-2c and 82% of patients staged N3.

The primary site of tumor also correlated with stage. Only 26% of patients with non-tonsil, non –base of tongue cancers had stage T1-or T2 disease. Limiting comparisons to the tonsil and base of tongue, there were differences between these 2 sites as well. Patients with base of tongue cancer were more likely to present with T4 primaries (20%) compared to those with tonsillar cancer (12%). Stage N0 was more common among patients with non-tonsil / non-base of tongue primaries (22%) than for those with cancers of the tonsil (5%) or base of tongue (4%). Stage N2c was also more common for patients with base of tongue cancer (24%) than for patients with tonsillar cancer (13%).

### Therapy

All patients had their cases discussed at a weekly multidisciplinary clinic and recommendations for therapy as well as assessment for treatment on protocol were made. During these years, we had participated in numerous therapy trials, and 210 patients in this cohort were treated on trial. Among the trials were 4 RTOG trials [8,13,21,22], the multiinstitutional phase III cetuximab trial [23] and 2 in-house phase I-II trials [16,24]. Final treatment decisions were made with the patient and their physicians. Pre-therapy gastrostomies were not mandated prior to therapy, and gastrostomy placement during therapy was individualized based on the clinical scenario.

**Figure 1:**
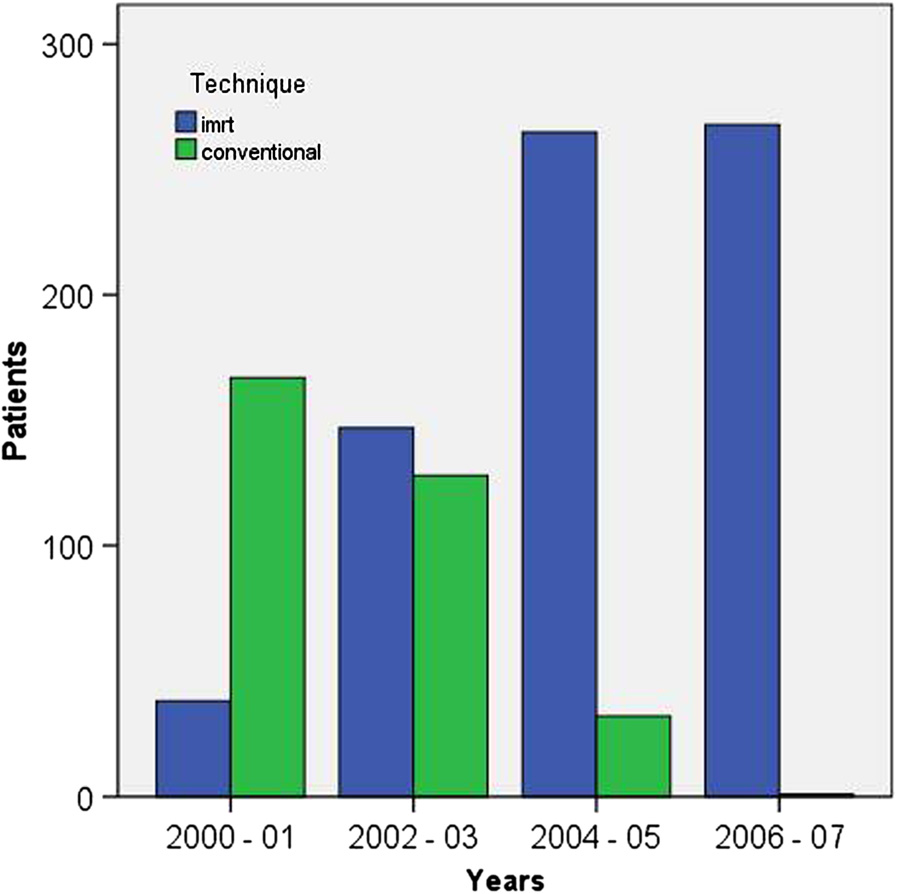
Number of patients treated with conventional radiation techniques and intensity-modulated radiation (IMRT).

IMRT was used to treat 69% of our patients. Figure 1 shows the use of IMRT over the years of study, as we began to incorporate IMRT into our practice in 2000; by 2006, it was used exclusively for our patients. While IMRT was more commonly used in never smokers, during the early 2000s many patients with T3 and T4 tumors were enrolled on studies not allowing the use of IMRT. Seven hundred patients were treated with once daily fractionation. The median dose was 70 Gy (2.2 – 75 Gy). Fourteen patients (1%) received less than 60 Gy. Nine of these 14 patients chose to discontinue treatment, 2 had treatment stopped due to toxicity and 3 died during therapy. The median number of fractions was 33 (1 – 44). Ipsilateral therapy was used to treat 66 patients (6%) with well- lateralized tonsillar cancer.

Systemic therapy was used in 645 patients (62%). Concurrent therapy was delivered to 513 patients (49%). Cisplatin was the most common concurrent drug (344 patients), followed by carboplatin (100 patients), and cetuximab (74 patients); 103 patients were treated with multidrug regimens. Two hundred forty-two (23%) patients were treated with neoadjuvant chemotherapy. All neoadjuvant regimens were platin and taxane based. One hundred twenty-four patients received both neoadjuvant and concurrent chemotherapy. There were no differences between delivering either concurrent or induction chemotherapy when grouped by smoking status. There were differences in the use of chemotherapy based on staging. The use of concurrent chemotherapy increased incrementally with T-category, as 14%, 39%, 80% and 90% of patients with T1,T2,T3,T4, respectively received concurrent chemotherapy. Induction therapy was more commonly used among patients with advanced nodal disease, as 34% of patients with N2b – N3 disease were treated with neoadjuvant therapy compared with only 5% of patients staged N0 – N2a. There was no difference in the use of induction chemotherapy based on T-category, as 23% and 23% of patients with T1-2 disease and T3-4 disease received neoadjuvant chemotherapy. Overall, 44% of patients with T1-2 disease received chemotherapy compared to 89% of patients with T3-4 disease.

Ninety-six patients had tonsillectomies prior to therapy. All 96 presented with lymphadenopathy. Twenty-eight of these 96 patients had tonsillectomies performed as part of their diagnostic staging procedures at MDACC. The remaining 68 presented to MDACC following tonsillectomy. Only one of these 68 patients had a tonsillectomy done as a therapeutic procedure for known malignancy. An additional 8 patients had excisions of their primary disease, and 3 had more comprehensive surgery on their primary tumor. Fifty-five patients had neck dissections and 88 had excisional biopsies of nodal disease prior to therapy. A total of 44 patients (4%) presented without obvious disease at both the primary site and neck.

Post-radiation neck dissections were performed in 253 patients; 64 patients (25%) had pathologic residual disease in their neck dissection specimens.

### Outcomes

The median follow-up time was 58 months (range, 1 – 130 months). Only 15 of 801 patients (2%) alive at last contact had less than 2 years of follow-up. The actuarial 5-year overall survival rate was 78% (95% CI, 75 – 81%).

Overall, 234 patients had disease recurrence, resulting in a 5-year recurrence free survival rate of 77% (95% CI, 74 – 80%). The 5-year local-regional control rate was 87% (95% CI, 85 – 89%), as 135 patients had disease recur at the primary site or in the neck (52 at the primary site, 35 in the neck, and 48 in both the primary and neck). The 5- year actuarial overall survival for patients with local-regional recurrence was 22% (95% CI, 15 – 30%).

The 5-year distant recurrence rate in patients who did not have local regional recurrence was 11% (95% CI, 9 – 13%).

Forty-five (5%) of patients with local regional control had a gastrostomy at last follow-up. Five of these patients also had tracheostomies. Increasing stage (both T and N), altered fractionation, conventional radiation technique, and concurrent chemoradiation were all associated with an increased rate of gastrostomy (Table 3, univariate analysis).

**Table 3:** Gastrostomy rates at last contact for patients with local-regional control

**Variable**	**Patient number**	**Gastrostomy at last follow-up**	***p*-value (chi-squared test)**
T-stage			<.01
1-2	604	2%	
3-4	307	10%	
N-stage			<.01
0−2a	338	3%	
2b – 2c	492	5%	
3	81	11%	
IMRT			<.01
Yes	657	3%	
No	254	9%	
Fractionation			<.01
Conventional	630	4%	
Altered	281	8%	
Induction Chemotherapy			.03
Yes	208	2%	
No	703	6%	
Concurrent Chemoradiation			<.01
Yes	422	7%	
No	489	3%	
Smoking			.4
Current	184	3%	
Former	338	5%	
Never	389	5%	

### Analysis of variables of interest

Primary site, smoking status, T-category, and radiation technique were all associated in multivariate analysis with local-regional control (Table 4). The crude local regional control rates for patients grouped by primary site were: tonsil 92%, base of tongue 86%, and other sites 68%. The 3 other variables were strongly correlated with each other. Eighty-one percent of patients with T1-2 disease were treated with IMRT, compared with 49% of patients with T3-4 disease; 75% of never smokers were treated with IMRT compared with 58% of current smokers; and 70% of never smokers had T1-2 disease, compared with 44% of current smokers.

**Table 4:** Local-regional control and survival

	**5-year local-regional control**	*p*- value*	**5-year overall survival**	*p*- value*
Age in years				
Continuous variable		.136		<.001
Sex		.896		.438
Male	87%		79%	
Female	87%		77%	
Race		.091		.261
White	88%		81%	
Non-white	76%		62%	
Smoke		.002		<.001
Current	75%		60%	
Former	88%		80%	
Never	92%		88%	
Primary site		.006		<.001
Tonsil	91%		83%	
Base of tongue	86%		80%	
Other	65%		43%	
T-category		<.001		<.001
1-2	94%		90%	
3-4	75%		60%	
N-category		.446		.001
0	74%		65%	
1 -2a	93%		86%	
2b – 2c	86%		79%	
3	80%		59%	
Lowest neck level		.529		.498
0	74%		67%	
2	90%		83%	
3	86%		77%	
4	76%		67%	
Treatment		.454		.027
XRT alone	92%		86%	
CTXRT	82%		72%	
Ind. CTX > XRT	89%		84%	
Ind. CTX > CTXRT	81%		71%	
Radiation technique		.003		.084
IMRT	92%		84%	
3D conformal	77%		66%	
Radiation Fractionation		.414		.150
Once- daily	90%		83%	
Altered fractionation	81%		70%	

*multivariable analysis; XRT – Radiotherapy; CTXRT – concurrent chemoradiation; Ind. CTX – Induction chemotherapy.

**Figure 2:**
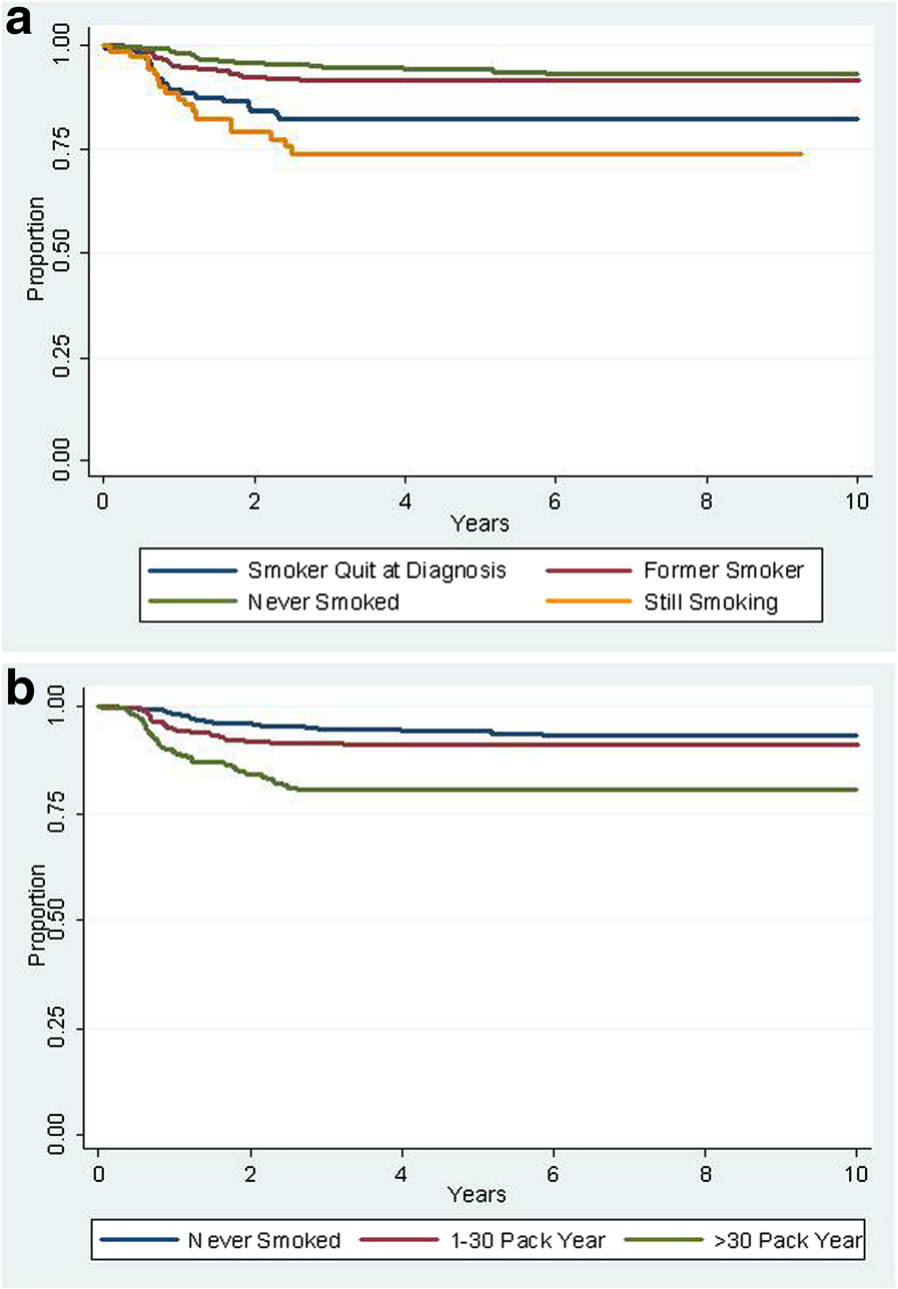
**Local-regional control rates stratified by smoking. a**) stratification by status; **b**) stratification by pack-year history.

The 5-year local-regional control rates were 75%, 88%, and 92% for current smokers, former smokers, and never smokers, respectively (Figure 2a). Among current smokers, the 5-year actuarial local regional control rates were 78% and 67% (p =.08) for those who quit smoking versus those who continued to smoke. While the number of years former smokers had quit and the number of pack-year among all smokers were both tested as continuous variables, only pack-year was significant. Evaluating smoking intensity (pack-year number) as a categorical variable (Figure 2b), the 5-year actuarial local-regional control rates were: zero pack-year, 92%, 1 – 30 pack-year, 89%, >30 pack year, 75% (p=.064 in multivariate analysis). Among current smokers, a lower smoking intensity was associated with quitting, as 44% of smokers who quit at diagnosis had 1–30 pack-year history, compared with only 27% of those who continued to smoke.

**Figure 3:**
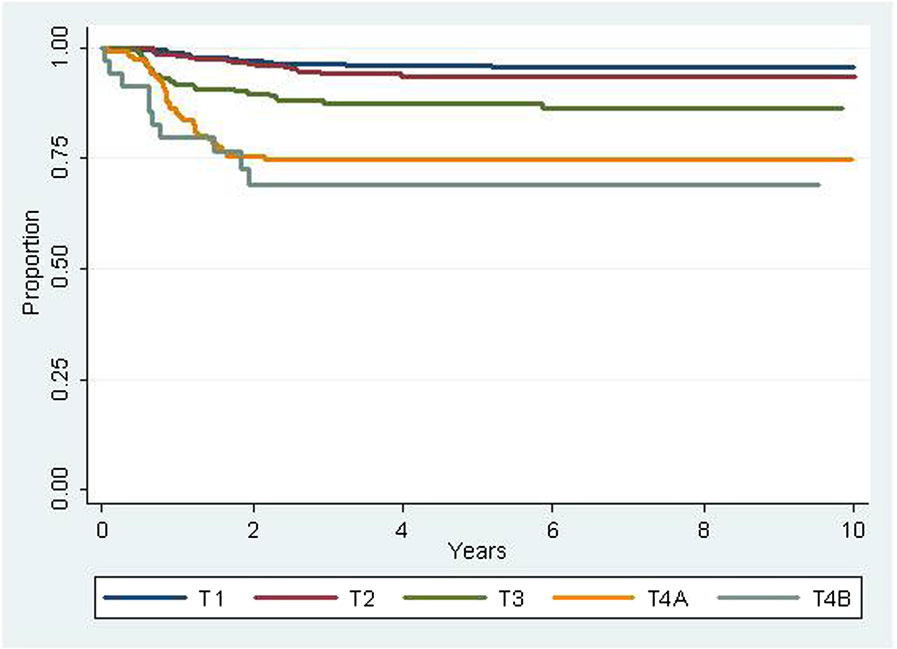
Local-regional control rates stratified by T-category.

The 5-year actuarial local-regional control rates for patients grouped by T-category were 96%, 93%, 85%, 65%, and 59% for stages T1, T2, T3, T4a and T4b, respectively (Figure 3). Differences were significant for the overall grouping, as well as for all paired comparisons except T1 vs. T2 and T4a vs. T4b.

The only treatment factor statistically significant for an association with local-regional control, was radiation treatment technique as those treated with IMRT had a 91% 5-year rate of local-regional control compared to 77% for patients treated with conventional techniques. However, significant confounding by T-stage exists. Subgrouping patients by T-category, the differences in local-regional control were only seen in patients staged T4, as those treated with IMRT had a 5-year control rate of 77% compared to 51% for those treated with conventional techniques.

**Figure 4:**
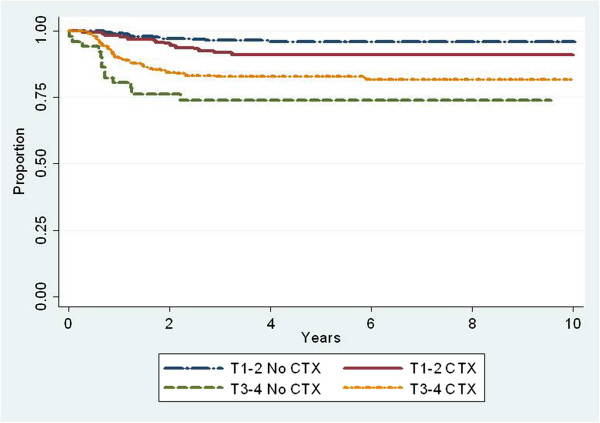
**Local-regional control rates stratified by use of concurrent chemotherapy (CTX) with radiation and T-category. **T1-2, no CTX, n = 470; T1-2, CTX, n = 170; T3-4 no CTX, n = 63; T3-4 CTX, n=343.

The use of concurrent chemotherapy was not associated with improved local-regional control. However in subgroup analysis, (Figure 4), patients with T3-4 disease who were treated with concurrent chemotherapy had a 5-year actuarial rate of local-regional control of 77% compared with 63% for those who did not receive concurrent chemotherapy (p=.01). Amongst patients with T1-2 disease, the 5-year actuarial rate of local-regional control was 91% for those treated with concurrent chemotherapy compared with 95% for those who did not receive concurrent chemotherapy (p=.06). In pairwise comparisons of concurrent single agent cisplatin, carboplatin and cetuximab, we did not find statistically significant differences in local-regional control.

N-category was not associated with local recurrence, but was associated with regional recurrence. The 5-year actuarial regional control rates were 96%, 90%, and 83% for stages N0-2a, N2b-c and N3, respectively. In multivariate analysis, disease recurrence in the neck (limited to patients with local control) was associated with nodal level, as regional control rates were progressively worse for patients with disease in levels 2, 3, and 4, respectively.

We further explored the interactions of T1-2 disease with N-category and chemotherapy on local-regional control, dichotimizing N-category into N1-2a and N2b-3. Thirty-nine (15%) T1-2, N1-2a patients, and 230 of T1-2, N2b-3 patients (60%) received chemotherapy (Table 5). No statistical improvement in local-regional control was seen for any of the groups who received chemotherapy, nor was any benefit seen when further subgrouped by induction or concurrent delivery.

**Table 5:** Local-regional control in patients with T1-2 disease dichotomized by N-category

	**T1-2, N1-2a**		**T1-2, N2b-c**	
	**Patient number**	**5-year local-regional control**	**Patient number**	**5-year local-regional control**
Treatment				
XRT alone	217	98%	154	92%
CTXRT	32	90%	87	90%
Ind. CTX > XRT	3	100%	96	94%
Ind. CTX > CTXRT	4	100%	47	93%

XRT – Radiotherapy; CTXRT – concurrent chemoradiation; Ind. CTX – Induction chemotherapy.

Analysis of distant failure restricted to patients with local-regional control revealed a higher metastasis rate seen with increasing age, higher T-category and lower neck levels. The 5-year distant failure rates were 7%, 16% and 25% for patients with disease in neck levels 2, 3 and 4, respectively. The 5-year distant failure rate for node negative patients was 13%. The 5-year distant failure rates for patients treated with and without induction chemotherapy were 13% and 11%, respectively (p =.636). Further subgroup analysis of patients staged N2b – 3 also showed no difference in distant failure rates for patients treated with and without induction chemotherapy (p = .642).

Multiple variables were statistically associated with survival (Table 4). N-category was associated with survival outcomes, but not in an orderly pattern, as patients with N2a disease had the best outcomes, and node negative patients did not have a higher survival rate than patients who were node positive. Current smokers, older patients, advanced T-category, primary site not in the tonsil or base of tongue, and the use of concurrent chemotherapy were associated with decreasing survival. The 5-year survival rates for patients treated with and without concurrent chemotherapy were 71% and 85%, respectively. Among patients with T1-T2 disease, the 5-year survival rates were 90% and 90% (p =.42) for those treated with and without concurrent chemotherapy, and for patients with T3-T4 disease the 5-year survival rates were 63% and 42% (p < .01) for those treated with and without concurrent chemotherapy.

Seventy-one (10%) patients who were recurrence free died of other causes. Death in these patients was associated (p<.01) with pack-years, as the 5-year survival rates for recurrence-free patients who were never smokers, smoked 1–30 pack-years and smoked >30 pack-years were 95%, 95% and 83%, respectively.

## Discussion

The management of oropharyngeal cancer continues to evolve with new developments in biology, technology, and clinical trials. Our current series describes a cohort of over 1000 patients with stage 3 and 4 oropharynx cancer treated over the past decade. High rates of disease control were achieved overall, though analysis of variables suggests a range of prognoses dependent principally on factors at presentation, which likely reflect the HPV status of these cancers [12]. However, it appears that treatment factors also influence outcomes, including the use of IMRT and chemotherapy in certain subgroups.

HPV status has recently been recognized as a major prognostic factor in outcomes of patients with oropharynx cancer [13]. However, the patients in our cohort were treated prior to routine testing for HPV (and p16), and retrospective pathologic review of over 1000 samples, many of which are from outside institutions was impractical for this review, and likely to result in a large amount of missing data points.

Our cohort did consist of 423 never smokers, and it is recognized that the vast majority of patients who are never smokers present with HPV positive tumors [25]. The 5-year local-regional control rate for these never smokers was 92% consistent with other reports on outcomes of HPV positive tumors. Without HPV status, it is more challenging to assess outcomes in HPV negative patients from our data, particularly since any history of tobacco exposure may confound the outcome of patients regardless of HPV status. Former smokers appeared to have similar outcomes to never smokers. This may be due to this cohort having a lesser intensity of smoking, but also, as noted recently by RTOG, [25] there is a greater percentage of HPV-positive tumors among former smokers, compared with current smokers.

Based on our past experiences, our general philosophy has been to use a risk-based approach that principally incorporates staging into our management algorithms. Historically our irradiated oropharyngeal cancer patients with T1 -2 disease had high rates of local control, [17,26,27] and patients who were node positive had high rates of regional control [28]. Thus, our decision to treat patients with concurrent chemoradiation was principally reserved for those patients with bulky primary disease (T3-4). Using this approach, we observed a clear benefit for our patients with T3-4 disease treated with concurrent chemoradiation. However, despite more intensive therapy based on T-category, primary tumor size and extent remained a strong prognostic factor. Regardless of smoking status, patients with more advanced disease had a greater probability of local recurrence. More advanced T-category was also associated with a higher likelihood of distant recurrence and poorer survival.

Despite the absence of HPV stratification, our 357 patients with T1-2 disease treated with radiation without concurrent chemotherapy had 95% and 90% 5-year local regional control and overall survival rates. Current guidelines favor concurrent therapy for all stage 3 and 4 patients. The evidence for this strategy, is robust, but based on numerous studies that included patients with the most advanced disease. Particularly for patients with T1 disease, data is sparse, as few randomized trials evaluating the role of concurrent therapy included patients with low volume disease [4]. The RTOG, for example, has excluded patients with T1 disease in their definitive trials of locally advanced head and neck cancer [2,13,21,22]. Prior to the routine use of chemotherapy for head and neck cancer, many had been critical of the AJCC and UICC stage grouping [29]; in particular these studies have demonstrated more favorable prognoses for patients with T1 node positive disease compared with other patients staged 3-4A. Thus, particularly for T1 staged patients (who typically represent about one-third of the oropharyngeal carcinoma population) we continue to favor radiation alone.

We could not demonstrate a benefit in outcome for patients treated with induction therapy, but as described above, the rates of distant disease have decreased suggesting a benefit at least compared to historical controls. Several years ago we reported on a cohort of node positive oropharyngeal carcinoma patients treated with radiation only [17]. These 299 patients had T1-2 disease and were treated without systemic therapy. In that report, the overall distant recurrence rate in patients with local regional control was 17%, compared with 11% for the entire current cohort here, and 7% for those staged T1-2. This observation is despite the current cohort having a higher percentage of patients staged N2b or greater, but is consistent with other recent reports of outcomes of patients treated with chemo-radiation for oropharyngeal cancer. Two principal differences between our earlier experience and the current series are that a greater percent of patients in the current series are likely HPV positive and the greater use of chemotherapy in the current experience. As many series describe similarities in distant metastases rates between HPV positive and negative patients [13,14], greater credence is given to the second hypothesis that chemotherapy likely impacted outcomes favorably.

The retrospective nature of this study prohibits category 1 based-evidence, and retrospective studies are commonly critiqued for inherent biases. We believe the magnitude of the cohort size obviates some of the concerns regarding the inferiority of a retrospective study. In particular, all 1046 patients were treated with radiation schedules designed for curative intent. Only 8 patients (< 1%) were excluded for being treated with palliative radiation schedules, and while the patient population was extracted from a database of irradiated patients, it was extremely rare for our multidisciplinary team to treat patients without radiation.

While MDACC is a tertiary cancer center, the population presented here is likely as representative of the general population presenting with oropharynx cancer. We did not include a comorbidity index of our patients, but the population did include patients who had comorbidities that sometimes either precluded the use of cisplatin, the drug with the strongest evidence of efficacy, or chemotherapy in general, thus including patients often excluded in phase 3 trials. Comorbidities, combined with biases of patients and individuals within a large multidisciplinary team impacted ultimate treatment decisions, and help explain that while we used a risk-based approach, still some of our patients with T1 category received chemotherapy, while 10% of our patients with T4-category did not receive chemotherapy.

We also chose a relatively narrow time frame (2000 – 2007) for this study, though going further back in time would have allowed us to expand the cohort significantly. This decision was made as we wanted to establish a robust trial with adequate follow-up (median, 58 months), but still reflect on modern treatment paradigms. Despite this goal, our series had great heterogeneity of therapy which reflects on the controversies of management for this disease. The last 2 decades saw great interest in treatment intensification to improve outcomes which ironically coincided with the increase in HPV related disease which has been demonstrated to be more chemotherapy and radiation sensitive [13-15,30]. Thus some patients were treated with chemotherapy doublets and radiation, while others received altered radiation fractionation. Favorable reports on taxane based induction regimens [5] led to an increased use of neoadjuvant chemotherapy, particularly for our patients with advanced nodal disease, though we frequently eliminated concurrent chemotherapy in these patients. Additionally, we often did not use chemotherapy in patients with small tumor burden despite having ‘advanced-staged’ disease. Radiation strategies changed, and we integrated more conformal radiation into our practice through the use of IMRT, and increased our use of ipsilateral radiation for selected patients with tonsillar cancer [31]. Even with this heterogeneity of patients and treatments, we report high rates of disease control and survival.

In conclusion, we describe a cohort of over 1000 oropharyngeal cancer patients with stage 3-4B disease irradiated over an 8 year period. These patients were treated in an era in which chemotherapy was becoming well integrated into the management of advanced head and neck cancer, and IMRT developed into the routine form of radiation planning and delivery. While several trials have demonstrated HPV positivity is associated with improved prognosis, we believe T-category, a classic “biomarker” remains paramount in treatment decision-making. Thus we still advocate radiation alone often as therapy for patients with low volume disease despite nominally being categorized as having stage 3 or 4 disease. We also caution against treatment deintensification for patients with T4 disease, even if biomarkers such as HPV status suggest a more favorable prognosis. While smoking status is not a consistent surrogate for HPV status, it is clear that never smokers have excellent outcomes. Furthermore, current smokers should be strongly encouraged, counseled, and treated for cessation as an important augmentation to the principal cancer treatment.

## Competing interests

The authors declare that they have no competing interests.

## Authors’ contributions

AG participated in the study design, recorded patient data, performed the statistical analysis and drafted the manuscript. MK, WM, RW, SF, KA, DR and EM participated in the acquisition of patient data and in drafting the manuscript. GG and BB participated in the drafting of the manuscript. All authors read and approved the final manuscript.
